# F32. DIFFERENCES BETWEEN YOUTH AT CLINICAL HIGH-RISK FOR PSYCHOSIS WHO DO NOT TRANSITION TO PSYCHOSIS: THE NORTH AMERICAN PRODROME LONGITUDINAL STUDY (NAPLS-2)

**DOI:** 10.1093/schbul/sby017.563

**Published:** 2018-04-01

**Authors:** Olga Santesteban-Echarri, Jacqueline Stowkowy, Scott W Woods, Lu Liu, Kristin S Cadenhead, Tyrone D Cannon, Barbara A Cornblatt, Thomas H McGlashan, Diana O Perkins, Larry J Seidman, Ming T Tsuang, Elaine F Walker, Carrie E Bearden, Daniel H Mathalon, Jean M Addington

**Affiliations:** 1 Hotchkiss Brain Institute, University of Calgary; 2 Yale University; 3 University of California San Diego; 4 Zucker Hillside Hospital; 5 University of North Carolina, Chapel Hill; 6 Harvard Medical School at Beth Israel Deaconess Medical Center, Massachusetts General Hospital; 7 University of California, San Diego, University of California, La Jolla; 8 Emory University; 9 University of California, Los Angeles; 10 University of California, San Francisco

## Abstract

**Background:**

In the clinical high risk (CHR) for psychosis literature, typically, the focus is on determining the risk of conversion to psychosis. However, between 70% and 85% of youth who meet CHR criteria do not develop psychosis during the follow-up period of the study in which they participate. The aim of this study is to focus on CHR youth who did not transition to psychosis and to determine whether there are differences amongst them.

**Methods:**

The North American Prodrome Longitudinal Study (NAPLS-2) is an 8-site prospective, longitudinal study including 764 help-seeking youth, age 12–35, meeting criteria for a psychosis risk syndrome based on the Structured Interview for Psychosis-risk Syndromes (SIPS), and 279 healthy controls (HC). For this analysis, only youth who did not make a transition to psychosis and completed 2 years of follow-up (n=278, 154 males, 124 females; mean age 18.8) were included. At the 24-month final assessment, the sample was divided into 3 groups: 1) those in remission, determined by scores ≤2 on all 5 attenuated psychotic symptoms on The Scale of Psychosis-risk Symptoms (SOPS); 2) symptomatic, determined by still having a rating of 3–5 on any one of the 5 attenuated psychotic symptoms on the SOPS; 3) prodromal progression, determined by continuing to meet the Criteria of Psychosis-risk Syndromes (COPS). The groups were compared at baseline and at 24-month follow-up on: age, gender, the presence of a current and lifetime psychiatric diagnosis, and social and role functioning. The use of antipsychotic medication was examined across all assessments (baseline, 6-, 12-, 18- & 24 months) using Generalized Linear Models to examine differences among the 3 groups.

**Results:**

Among the participants, 110 (39.57%) were in-remission, 93 (33.45%) symptomatic, and 75 (26.98%) prodromal progression. At baseline there were no significant differences in age, gender, social and role functioning, or SCID diagnoses except on current PTSD (p=.001) with most cases in the prodromal progression group, and on current anxiety disorder (p=≤.0001) with most cases in the symptomatic group. The prodromal progression had significantly higher ratings on unusual thought content compared to the in-remission group and significantly higher ratings on suspiciousness than the symptomatic group. At 24-month follow-up there were significant differences in negative symptoms (p=≤.0001) between prodromal progression (M=9.19), symptomatic (M=8.84), and in remission (M=5.99) groups; and social functioning (p=≤.005; M=6.56, M=6.68, M=7.20 respectively). Although the in-remission group had the highest ratings on social functioning these were significantly lower in social (M=7.20) and role (M=6.68) functioning than HC (M=8.73, M=8.62 respectively). The groups did not differ on their use of antipsychotics over the course of their 2 years in the study.

**Discussion:**

There were very few differences on baseline measures amongst the different two-year outcome groups. At 2 years, even though those in remission had improved social and role functioning relative to the other 2 groups, they still had lower social and role functioning than HC.

